# Speech paired vagus nerve stimulation restores neural sound processing in a rat model of autism

**DOI:** 10.3389/fnins.2025.1600024

**Published:** 2025-06-13

**Authors:** Brendan M. Williams, Yuko Tamaoki, Tanya T. Danaphongse, Isabella K. Myers, Samantha L. Kroon, Maria P. Solano, Allan A. Jacob, Jonathan R. Riley, Min Chen, Seth A. Hays, Crystal T. Engineer

**Affiliations:** ^1^Department of Neuroscience, School of Behavioral and Brain Sciences, The University of Texas at Dallas, Richardson, TX, United States; ^2^Texas Biomedical Device Center, The University of Texas at Dallas, Richardson, TX, United States; ^3^Department of Mathematical Sciences, University of Texas at Dallas, Richardson, TX, United States; ^4^Department of Bioengineering, Erik Jonsson School of Engineering and Computer Science, University of Texas at Dallas, Richardson, TX, United States

**Keywords:** valproic acid, vagus nerve stimulation, speech, anterior auditory field, autism spectrum disorder, preclinical research, synaptic plasticity, neuromodulation

## Abstract

**Introduction:**

Prenatal exposure to valproic acid (VPA) is a common environmental cause of autism spectrum disorder (ASD) and often leads to expressive and receptive language impairments. Similar communication difficulties among individuals with ASD are often linked to abnormal subcortical and cortical sound processing. Rodents prenatally exposed to VPA exhibit degraded cortical responses to speech and an impaired ability to behaviorally discriminate speech sounds.

**Methods:**

We sought to determine whether sound processing could be restored with paired vagus nerve stimulation (VNS). In a first experiment, we evaluated whether sound-paired VNS would alter *in vivo* extracellular multi-unit responses to tones, noise burst trains, and speech sounds from the anterior auditory field. We next sought to evaluate whether improvements to neural sound processing led to improvements in sound discrimination ability. In a second experiment, rats underwent go/no-go sound discrimination testing where VNS was paired with successful trials.

**Results:**

We found that VPA-exposed rats had degraded spectral, temporal, and speech sound processing compared to saline-exposed control rats. VPA-exposed rats which received sound-paired VNS exhibited a partial or full restoration of processing across sound types. However, across several sound discrimination tasks, we did not observe changes in behavioral performance in response to prenatal exposure to VPA or VNS.

**Discussion:**

Our study is the first to show that speech-paired VNS leads to a generalized improvement in cortical sound processing across sound types, rescuing neural processing among VPA-exposed rats. These results provide a framework for future studies to develop VNS-based interventions for communication disorders.

## 1 Introduction

For individuals with autism spectrum disorder (ASD), communication difficulties can pervade everyday life. Many children with ASD have difficulties with receptive language (perception) and expressive language (production) (Charman et al., 2003; Loucas et al., 2008; Russo et al., 2008, 2009; Matsuzaki et al., 2019). These impairments in language ability are correlated with weaker and delayed cortical and subcortical responses to sounds (Rosenhall et al., 2003; Alcántara et al., 2004; Russo et al., 2009; Otto-Meyer et al., 2018; Matsuzaki et al., 2019; Ramezani et al., 2019; Seif et al., 2021). Physiological alterations to sound processing can make tracking rapid spectrotemporal changes difficult, impairing speech perception (Paul et al., 2005; Globerson et al., 2015). Restoring sound processing could lead to improvements in sound perception.

The current gold standard intervention for ASD, Early Intensive Behavioral Intervention (EIBI), leads to meaningful improvements in speech processing (Frazier et al., 2021). However, outcomes are highly variable, and a large portion of participants (~40%) do not reach normative levels after years of intensive treatment (Klintwall et al., 2015; Frazier et al., 2021). The development of an adjunct to traditional therapy may improve outcomes and accelerate treatment (Anderson et al., 2022).

Stimulation of the vagus nerve is a potential adjunctive therapy for enhancing the effectiveness of traditional rehabilitation therapy (Engineer et al., 2017). When stimulated, the vagus drives activity in the locus coeruleus, nucleus basalis, and the dorsal raphe nucleus (Hulsey et al., 2016, 2019; Bowles et al., 2022; Martin et al., 2024). The resulting efflux of norepinephrine, acetylcholine, and serotonin to the surrounding cortices can drive cortical plasticity that is specific to a temporally paired stimulus or movement (Borland et al., 2016, 2019; Hulsey et al., 2016, 2019; Buell et al., 2018, 2019; Morrison et al., 2019). Sound-paired vagus nerve stimulation (VNS) causes a reorganization of the auditory cortex to increase the representation of the paired sound frequency and can alter the receptive field properties of cortical neurons (Engineer et al., 2011; Shetake et al., 2012; Borland et al., 2016, 2019; Buell et al., 2018; Adcock et al., 2020b). In animal models with auditory processing dysfunction, including rats with tinnitus and rats with *Mecp2^+/-^* mutation, VNS-sound pairing has been shown to restore both sound processing and sound perception, resulting in improved neural and behavioral outcomes (Engineer et al., 2011; Adcock et al., 2020b). This suggests that VNS has the potential, as an adjunctive therapy, to reverse physiological deficits in auditory processing.

Prenatal exposure to the anticonvulsant sodium valproate (VPA), a widely recognized environmental cause of autism, alters sound processing across the auditory pathway in humans and rodents. Humans prenatally exposed to VPA (fetal valproate syndrome) have receptive and expressive language impairments (Nadebaum et al., 2011; Christensen et al., 2013). Rodents prenatally exposed to VPA exhibit degraded sound processing across subcortical and cortical structures (Engineer et al., 2014a, 2014b; Anomal et al., 2015; Cheng et al., 2022; Tamaoki et al., 2024). For VPA-exposed rodents, these impairments in sound processing are correlated with impairments in behavioral discrimination of temporal rates (Cheng et al., 2022) or speech sounds (Engineer et al., 2014b). It is possible that VNS-sound pairing could be used to rescue the weakened and delayed cortical responses to speech sounds and the impaired behavioral discrimination of speech sounds observed among VPA-exposed rats (Engineer et al., 2014a, 2014b; Tamaoki et al., 2024).

Although neural sound processing and behavioral sound discrimination are often closely related, it is not known whether restoring neural sound processing will improve behavioral sound discrimination among VPA-exposed rats. If this is the case, VNS-sound pairing may represent a clinically feasible strategy for restoring speech processing and communication among individuals with ASD.

To directly test this hypothesis, we first evaluated whether VNS paired with speech sounds would alter sound processing in the auditory cortex of VPA-exposed rats. We subsequently tested whether delivering VNS during an auditory discrimination task would improve sound discrimination ability.

## 2 Materials and methods

### 2.1 Animals

All procedures were in accordance with The University of Texas at Dallas’ Institutional Animal Care and Use Committee protocol #18-07. Experiments were conducted in male and female Sprague Dawley rats (*n* = 56; 3–6 months old) from 23 dams. Founding pairs were ordered from Charles River Laboratories (Wilmington, MA) and offspring were bred in-house at the UT Dallas vivarium facility (Engineer et al., 2014a, 2014b). Rats were single housed in a reverse 12:12 light–dark cycle. During behavior training, animals were food restricted on weekdays with ad libitum access to food on weekends, maintaining a minimum 85% body weight.

### 2.2 Model

Rats were prenatally exposed to either sodium valproate (600 mg/kg body weight; Sigma Aldrich; St. Louis, Mo. product # P4543) dissolved in physiological sodium chloride (saline), or 1 mL of saline alone delivered through intraperitoneal injection to the pregnant dam on embryonic day 12.5 (Schneider and Przewłocki, 2005; Kim et al., 2011; Engineer et al., 2014a, 2014b; Anomal et al., 2015; Tamaoki et al., 2024).

### 2.3 Acoustic stimuli

Speech sounds were spoken by a single native-English speaking female and shifted up an octave into the rat hearing range using the STRAIGHT Vocoder (Kawahara, 1997; Engineer et al., 2008, 2011, 2014a, 2014b; Tamaoki et al., 2024). These sounds are approximately 500 ms in duration and were calibrated so the loudest 100 ms of the sound is presented at 60 dB SPL. Spectrograms, amplitude envelopes, and power spectrums for the speech sounds used in this study have been previously reported (Engineer et al., 2008, 2015). Tones and noise bursts played during in-vivo electrophysiology were generated with Tucker-Davis Technologies (TDT; Alachua, FL) SigGen signal generator and calibrated to varying frequencies and intensities with TDT SigCal signal calibrator.

### 2.4 Vagus nerve surgery

After postnatal day 90, rats underwent cuff and headcap implantation (Borland et al., 2016, 2019, 2023; Buell et al., 2018, 2019; Rios et al., 2019; Adcock et al., 2020b; Bucksot et al., 2020). Following initial induction using 2 mL of isoflurane, a VetFlo^tm^ Vaporizer Single Channel Anesthesia System (Kent Scientific; Torrington, CT) was used to sustain isoflurane delivery and maintain anesthesia for the duration of surgery. Vitals were monitored using MouseOx Plus Oximeter for Rodents (STARR Life Sciences; Oakmont, PA) and body temperature was maintained at 37°C. A custom-made Teflon-coated platinum-iridium bipolar cuff electrode was fitted around the left cervical vagus nerve (Rios et al., 2019). Lead wires were run subcutaneously from the electrode to an omnetics headcap connector fixed to the skull with stainless steel bone screws and cemented with acrylic. The function of the cuff was confirmed using the Hering-Breuer reflex (Bucksot et al., 2020). Following surgery, a triple antibiotic ointment was applied to incision sites. Animals received 10 mL of Dextrose Ringers injected subcutaneously to maintain hydration, and one 2 mg tablet of both Enrofloxacin (Baytril) and Carprofen (Rimadyl) (Bio-Serv; Flemington, NJ) to prevent infection and reduce inflammation. Postoperative care was repeated for the three days following surgery, and animals recovered for at least one week prior to resuming behavior or VNS-pairing. All surgical procedures replicate those described previously (Borland et al., 2016, 2019, 2023; Buell et al., 2018, 2019; Rios et al., 2019; Adcock et al., 2020b; Bucksot et al., 2020).

### 2.5 Experiment 1: neurophysiological recordings

Male and female rats (SAL-exposed *n* = 10, VPA-exposed *n* = 10, and VNS-paired VPA-exposed *n* = 8) underwent in-vivo multi-unit extracellular recording from anterior auditory field (AAF).

#### 2.5.1 Sound-paired VNS

A subset of VPA-exposed rodents (*n* = 8) underwent 20 days of VNS-sound pairing prior to electrophysiological recording. Previous work has varied the duration, pulse width, frequency, and intensity of vagus nerve stimulation to identify optimal parameters for inducing synaptic plasticity (Borland et al., 2016; Buell et al., 2018, 2019; Loerwald et al., 2018). Based on their findings, we delivered 500 ms, 100 μs biphasic 16 pulse, 30 Hz, 0.8 mA VNS paired with the speech sound “dad” in a double-walled sound attenuated booth. The onset of VNS preceded the onset of the speech sound by 50 ms. Pairings were randomly interleaved with silence trials for an average intertrial interval of 30 s, and stimulation was delivered via A-M Systems Isolated High Power Stimulators (Model 4100; Sequim, WA). Rats received 300 VNS-sound pairings per 2.5 h session. These methods are identical to our previous publications (Engineer et al., 2011; Borland et al., 2016, 2019, 2023; Buell et al., 2018).

#### 2.5.2 Anterior auditory field electrophysiology

Multi-unit extracellular activity was recorded from 357 sites across 10 saline (SAL)-exposed rats, 330 sites across 10 VPA-exposed rats, and 285 sites across 8 VNS-paired VPA-exposed rats. Rats were anesthetized for recordings with sodium pentobarbital (50 mg/kg body weight) and supplemented with dilute pentobarbital (8 mg/kg body weight) as needed. Vitals were monitored using MouseOx Plus Oximeter for Rodents (STARR Life Sciences; Oakmont, PA) and body temperature was maintained at 37° C. Prior to recording, rats received a tracheotomy and cisterna drain to ease breathing and reduce brain swelling. A cranial window was opened and the dura was resected to expose right AAF. Two 2×1 Parylene-coated tungsten microelectrodes (FHC, 1–2 MΩ) were lowered into layer 4/5 (~600 μm) of the cortex, and a speaker was placed 10 cm from the left ear. A stimulus set consisting of 25 ms tone pips ranging in frequency from 1-32 kHz and in intensity from 0-75 dB SPL, speech sounds, and noise burst trains (six 25 ms bursts, at a rate of 7.5, 10, 12.5, or 15 Hz) were presented (Engineer et al., 2014a, 2014b; Borland et al., 2016, 2019, 2023; Buell et al., 2018, 2019; Adcock et al., 2020b; Tamaoki et al., 2024). Speech sounds (“dad,” “bad,” “gad,” “tad,” “sad,” “rad,” and “lad”) and noise burst trains were pseudo-randomly repeated 20 times each. Neural responses passed through an RA16 preamplifier and were recorded with BrainWare (TDT). AAF was identified based on its reversed tonotopy compared to primary auditory cortex (A1) (with low frequency anterior and high frequency posterior) and fast responses (10-15 ms onset latency) (Polley et al., 2007). Similarly, a reversal in tonotopy, the loss of tuning or response strength, delay of response latency, and monotonicity was used to border AAF (Polley et al., 2007; Centanni et al., 2013; Engineer et al., 2014a, 2014b; Shi et al., 2019).

#### 2.5.3 Data analysis

Utilizing responses to tones, we characterized receptive field properties at each recording site, including: the characteristic frequency; the lowest threshold (dB SPL) tone to evoke a response at the characteristic frequency; the bandwidth of neuron tuning at 10-40 dB SPL above the threshold; response onset and peak latency at 60 dB SPL; the percentage of recording sites responding to tone frequencies within each of 5 one-octave frequency bins (1-2, 2-4, 4-8, 8-16, and 16-32 kHz); the number of spikes evoked per tone within +/− a half octave from the characteristic frequency of the recording site; and the rate level function. For recordings to speech sounds, we compared the average driven response to the onset of the consonant (1-40 ms), response onset and peak latency, and neural classifier accuracy between groups. For recordings to noise bursts, we compared average firing rate, steady state responses to alternating bursts, response onset latency, Rayleigh statistic, vector strength, and paired-pulse ratio between groups. Steady state responses to noise burst trains were calculated by averaging the driven firing rate from bursts three onwards (366-433 ms depending on repetition rate) (Regan, 1966). Rayleigh statistic was used to describe response uniformity across time in a circular space. Recording sites with a Rayleigh statistic above 13.8 were considered phase locked since responses deviated from uniformity in synchrony with the stimulus. Vector strength was used to describe the degree of separation from uniform (i.e., the quality of the deviation) (Pandya et al., 2008; Shetake et al., 2012). Paired-pulse ratio was calculated by dividing the driven response to the second noise burst in the train by the first (P2/P1) (Debanne et al., 1996).

Because the data contained multiple levels of hierarchical nested data with an unequal number of repeated measures (e.g., recording sites), we analyzed the data with linear mixed models in R (v. 4.3.3 – 4.4.0) and RStudio (v. 2024.4.1) (Bolker et al., 2009; Pinheiro and Bates, 2009). Data was tested for normality with the Shapiro–Wilk or Shapiro-Francia test depending on its kurtosis (Mbah and Paothong, 2015). In cases where the response variable was normally distributed, the Lme4 package (v. 1.1-35.3) was used for linear mixed effect models (Bates et al., 2015). In cases where the data was not normally distributed, the glmmTMB package (v. 1.1.9) was used for generalized linear mixed effects models (glmm) (Brooks et al., 2017). The response variable (e.g., number of action potentials, rate of action potential firing, response bandwidth, latency to respond, etc.) was dependent and compared across the fixed effect of experimental group. Where applicable, the model was updated to include additional fixed effects and interactions (e.g., tone intensity, tone frequency, noise burst repetition rate). Animal and recording sites were treated as random effects, were nested, and had a fixed intercept and a random slope. When data was non-normally distributed, family was selected based on the distribution of the data. Since our response variable was neuronal activity, the Tweedie family with a log link was used for most of the analysis (Moshitch and Nelken, 2014). In cases where the data was non-normally distributed, not continuous, and did not include negative numbers or zero inflation, Beta Family and Binomial distributions were used. Akaike information criterion (AIC), Bayesian information criterion (BIC), R-squared, covariance structure, and residual diagnostics were used to compare model fit while avoiding overfitting the data. Once an appropriate model was selected, Type II Wald Chi-square tests were performed to determine if fixed effects were significant predictors of the response variable (car package v. 3.1.2) (Fox and Weisberg, 2019). Following a significant main effect, Tukeys corrected pairwise comparisons were performed with the emmeans package (v. 1.10.1) (Lenth et al., 2018). Data was visualized with GraphPad Prism (v. 10.2.3) and Matlab (v. 2022a).

### 2.6 Experiment 2: go/no-go auditory tasks

Male rats (SAL-exposed *n* = 10, VPA-exposed *n* = 10, and VNS-paired VPA-exposed *n* = 8) were trained to discriminate speech sounds using a go/no-go operant training task.

#### 2.6.1 Pretraining

During the first stage of training, rats learned to nose-poke for a 45 mg nutritionally complete sugar pellet (Bio-Serv; Flemington, NJ). After independently poking for 100 pellets per session for two sessions, rats learned to nose-poke when they detected the target word and refrain from poking during silence catch trials. As they progressed through the training stages, the hit-window for correct nose-pokes decreased from 4 s to 3 s. If the animal poked during a silence catch trial or >3 s after the sound presentation, it received a 6 s “timeout” during which the booth lights shut off and pokes elicited no feedback. Rats completed an average of 17 (± 3 SD) one-hour sessions of training on detection until they could reliably identify the target from silence catch trials, reaching a performance criterion of 75% correct for four sessions. After reaching proficiency, they were removed from training for cuff implantation surgery (Engineer et al., 2013, 2014b; Carroll et al., 2024).

#### 2.6.2 Speech sound discrimination tasks

After recovery from cuff and headcap implantation, rats began a series of sound discrimination tasks where they learned to discriminate the target sound “dad” from similar non-target sounds differing by initial consonant: “bad,” “gad,” “tad,” and “sad” (Engineer et al., 2013, 2014a, 2014b; Adcock et al., 2020a; Carroll et al., 2024). A subset of VPA-exposed rats (*n* = 8) received success-paired VNS during this task (Bowles et al., 2022). After 20 days of training with two sessions per day, all rats advanced to increasingly complex discrimination tasks involving multiple speakers, sounds that were truncated, compressed, or presented in noise (Engineer et al., 2013; Adcock et al., 2020a). These tasks are described in detail in the Supplementary Figures legend.

#### 2.6.3 Success-paired VNS

During the first sound discrimination task, VNS was triggered with pellet delivery after nose-poking to the target sound. The total number of stimulations was dependent on the animal’s performance and was on average 92 (± 28 SD) per session.

#### 2.6.4 Data analysis

Behavioral performance was quantified as percent correct. Data was tested for normality with Shapiro–Wilk or Shapiro-Francia depending on its kurtosis (Mbah and Paothong, 2015). All behavioral data was normally distributed and analyzed with Bonferroni corrected two-way repeated measures ANOVA. Data was analyzed and visualized with GraphPad Prism (v. 10.2.3).

## 3 Results

### 3.1 Experiment 1: neurophysiological recordings

Since speech processing is dependent on segregating distinct patterns of neural activity, and AAF has a previously established role in pattern discrimination (Lomber and Malhotra, 2008), it is likely that AAF plays a role in discriminating between speech sounds. In a previous study, AAF and not A1 was specifically impaired at processing speech sounds in rodents prenatally exposed to VPA (Engineer et al., 2014a). Utilizing extracellular multi-unit recordings from AAF, this experiment has the goal of documenting changes to how sound is processed following prenatal exposure to VPA and postnatal sound-paired VNS.

#### 3.1.1 Receptive field properties

**Figure 1:**
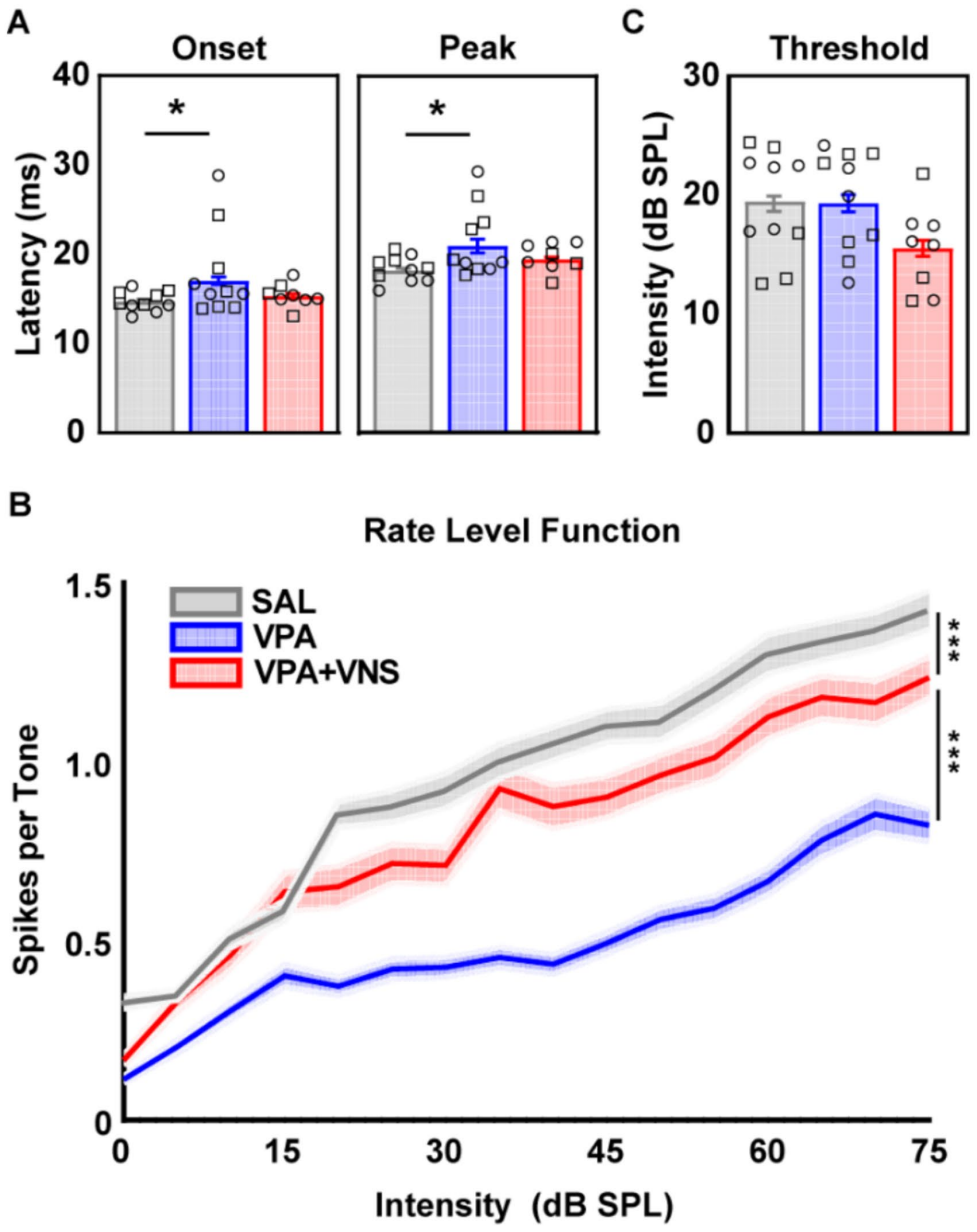
Degraded response latency and intensity coding partially restored with VNS. Data represent responses to tone pips within +/− a half octave from the characteristic frequency of the recording site. **(A)** Onset and Peak of response latency to 60 dB SPL tone pips. **(B)** Average response to tone pips varying in intensity. The line represents the mean and shading represents the SEM of recording sites. **(C)** Average response threshold to tone pips. Detailed group N and information on post hoc testing is available in Table 1 and Supplementary Table 2. Bars represent mean and standard error of the mean (SEM) for recording sites. Individual data points represent the mean across all AAF recording sites for a single animal. Shape of individual data points denote sex; squares are male and circles are female (* = *p* < 0.05, *** = *p* < 0.001).

Receptive field properties describe the sensitivity and selectivity of neurons to simple sound characteristics. A failure to encode simple sound characteristics (e.g., frequency or loudness) could lead to widespread processing impairments of complex sounds. To quantify receptive field properties, we recorded AAF responses to tone pips ranging from 1-32 kHz frequency and 0-75 dB SPL intensity. When we compared the latency of response onset to each tone, there was a main effect of group (χ2 = 6.41, df = 2, *p* = 0.04) and post hoc comparisons revealed a significant delay in response onset among VPA-exposed rats compared to SAL-exposed rats. This delay was partially rescued in VNS-paired VPA-exposed rats who were no longer significantly different from SAL-exposed controls or untreated VPA-exposed rats (Figure 1A; Table 1). Likewise, when peak response latency was compared, a significant main effect of group was observed (χ2 = 6.79, df = 2, *p* = 0.03) and post hoc comparisons revealed a significant delay in peak of response among VPA-exposed rats which was partially restored among VNS-paired VPA-exposed rats (Figure 1A; Table 1). We next investigated whether response strength was altered as a function of sound intensity since children with ASD often exhibit hypo or hypersensitivity to sounds, which is often intensity dependent. When comparing response strength across sound intensity, we observed robust differences in response strength across intensities, with main effects of both group (χ2 = 105, df = 2, *p* < 0.0001) and intensity (χ2 = 6,017, df = 15, *p* < 0.0001), and group x intensity interaction (χ2 = 194, df = 30, *p* < 0.0001). Post hoc comparisons revealed significant between group differences at each intensity level, with VPA-exposed rats responding significantly weaker than SAL-exposed controls and VNS-paired VPA-exposed controls. VNS-sound pairing partially restored response strength across intensity levels, responding significantly stronger than untreated VPA-exposed rats, but in most cases still significantly weaker than SAL-exposed rats (Figure 1B; Supplementary Table 2).

**Table 1:** Model and estimated marginal means for Figures 1–4.

Figure	Model	Groups (*n* sites/*n* animal)	Back transformed estimated marginal mean	Lower CI	Upper CI	SE	Contrast	*P* value
Figure 1A (onset)	GLMM: Onset ~ Group + Sex + Group * Sex + (1 | Animal/Channel), family = Tweedie (link = “log”)	Saline (*n* = 357/10)	14.4	13.2	15.6	0.61	SAL/VPA	**0.02**
		VPA (*n* = 330/10)	16.9	15.5	18.4	0.74	SAL/VPA + VNS	0.83
		VNS (*n* = 287/8)	14.9	13.5	16.4	0.73	VPA/VPA + VNS	0.15
Figure 1A (peak)	GLMM: Peak ~ Group + Sex + Group * Sex + (1 | Animal/Channel), family = Gamma (link = “sqrt”)	Saline (*n* = 357/10)	18.3	17.1	19.5	0.6	SAL/VPA	**0.02**
		VPA (*n* = 330/10)	20.7	19.4	22	0.72	SAL/VPA + VNS	0.47
		VNS (*n* = 287/8)	19.4	18	20.8	0.67	VPA/VPA + VNS	0.38
Figure 1B*	GLMM: Spikes ~ Group + Sex + Intensity + Group * Intensity + (1 | Animal/Channel), family = Tweedie (link = “log”)	Saline (*n* = 357/10)	0.66	0.59	0.75	0.04	SAL/VPA	**<0.0001**
		VPA (*n* = 330/10)	0.25	0.22	0.29	0.01	SAL/VPA + VNS	**<0.0001**
		VNS (*n* = 287/8)	0.46	0.4	0.52	0.03	VPA/VPA + VNS	**<0.0001**
Figure 1C	GLMM: Threshold ~ Group + Sex + Group * Sex + (1 | Animal/Channel), family = Tweedie (link = “log”)	Saline (*n* = 357/10)	18.6	16.1	21.6	1.41	SAL/VPA	0.97
		VPA (*n* = 330/10)	19	16.2	17.8	1.53	SAL/VPA + VNS	0.09
		VNS (*n* = 287/8)	14.9	12.5	17.8	1.35	VPA/VPA + VNS	0.06
Figure 2C*	GLMM: PL ~ Group + NB + Sex + Group * NB + Group * Sex + (1 | Animal/Channel), family = binomial	Saline (*n* = 357/10)	11.1^&^	9.73	12.5	0.71	SAL/VPA	**0.02**
		VPA (*n* = 330/10)	9.32^&^	8.2	10.4	0.56	SAL/VPA + VNS	0.52
		VNS (*n* = 287/8)	10.24^&^	8.81	11.7	0.73	VPA/VPA + VNS	0.39
Figure 2D*	GLMM: VS1 ~ Group + NB + Sex + Group * NB + Group * Sex + (1 | Animal/Channel), family = Tweedie (link = “log”)	Saline (*n* = 357/10)	0.75	0.71	0.79	0.01	SAL/VPA	0.11
		VPA (*n* = 330/10)	0.7	0.66	0.73	0.01	SAL/VPA + VNS	0.81
		VNS (*n* = 287/8)	0.73	0.69	0.77	0.02	VPA/VPA + VNS	0.41
Figure 2E*	GLMM: PPR ~ Group + NB + Sex + Group * NB + Group * Sex + (1 | Animal/Channel), family = Tweedie (link = “log”)	Saline (*n* = 357/10)	0.49	0.39	0.63	0.06	SAL/VPA	**0.008**
		VPA (*n* = 330/10)	0.84	0.65	1.07	0.1	SAL/VPA + VNS	0.89
		VNS (*n* = 287/8)	0.54	0.41	0.71	0.07	VPA/VPA + VNS	**0.05**
Figure 3B (onset)	GLMM: Onset ~ Group + Sex + Group * Sex + (1 | Animal/Channel), family = gaussian (link = “log”)	Saline (*n* = 357/10)	18.8	18	19.7	0.44	SAL/VPA	0.91
		VPA (*n* = 330/10)	19.1	18.2	20	0.46	SAL/VPA + VNS	0.82
		VNS (*n* = 287/8)	19.2	18.2	20.3	0.52	VPA/VPA + VNS	0.97
Figure 3B (peak)	GLMM: Peak ~ Group + Sex + Group * Sex + (1 | Animal/Channel), family = gaussian (link = “log”)	Saline (*n* = 357/10)	23.9	22.4	25.6	0.82	SAL/VPA	0.42
		VPA (*n* = 330/10)	25.5	23.7	27.3	0.92	SAL/VPA + VNS	0.88
		VNS (*n* = 287/8)	24.5	22.7	26.5	0.96	VPA/VPA + VNS	0.75
Figure 3C	GLMM: Spikes ~ Group + Sex + Group * Sex +(1 | Animal/Channel), family = gaussian (link = “identity”)	Saline (*n* = 357/10)	1.3	1.18	1.42	0.06	SAL/VPA	**0.02**
		VPA (*n* = 330/10)	1.07	0.95	1.2	0.06	SAL/VPA + VNS	0.09
		VNS (*n* = 287/8)	1.49	1.35	1.63	0.06	VPA/VPA + VNS	**<0.0001**
Figure 3D	GLMM: Spikes ~ Group + Sex + Group * Sex +(1 | Animal/Channel), family = Tweedie (link = “log”)	Saline (*n* = 357/10)	1.69	1.44	1.97	0.13	SAL/VPA	0.94
		VPA (*n* = 330/10)	1.75	1.49	2.06	0.14	SAL/VPA + VNS	**0.0004**
		VNS (*n* = 287/8)	2.67	2.24	3.18	0.24	VPA/VPA + VNS	**0.001**
Figure 3E	GLMM: Spikes ~ Group + Sex + Group * Sex +(1 | Animal/Channel), family = Tweedie (link = “log”)	Saline (*n* = 357/10)	3.33	2.97	3.73	0.19	SAL/VPA	0.55
		VPA (*n* = 330/10)	3.06	2.71	3.44	0.18	SAL/VPA + VNS	**0.001**
		VNS (*n* = 287/8)	4.55	4	5.17	0.29	VPA/VPA + VNS	**<0.0001**
Figure 4A	GLMM: PerCor ~ Group + Sex + Group * Sex + (1 | Animal/Channel), family = beta_family (link = “probit”)	Saline (*n* = 357/10)	0.69	0.66	0.71	0.01	SAL/VPA	**0.02**
		VPA (*n* = 330/10)	0.64	0.61	0.67	0.01	SAL/VPA + VNS	0.88
		VNS (*n* = 287/8)	0.7	0.67	0.73	0.01	VPA/VPA + VNS	**0.01**

* Results shown here are averaged across a factor, for all data see Supplementary Tables. & Results shown here are linear predictor (log-odds) not back-transformed since back-transforming a binomial model results in a probability rather than an estimated marginal mean. Bolded *p* values are statistically significant.

Improvements in response latency and strength among VNS-paired VPA-exposed rats arose without changes to the sensitivity or selectivity of cortical neurons. When comparing response threshold, there was a main effect of group (χ2 = 6.48, df = 2, *p* = 0.03), but post hoc comparisons revealed no significant between group differences (Figure 1C; Table 1). Across all receptive field bandwidths we observed no main effects, suggesting no differences in neuron frequency tuning between groups (Supplementary Figure 1A; Supplementary Table 1). Next, utilizing the characteristic frequency of recording sites, we calculated the percentage of recording sites for each animal tuned to frequencies within each of 5 one-octave frequency bins (1-2, 2-4, 4-8, 8-16, and 16-32 kHz). There was a main effect of frequency octave (χ2 = 269, df = 4, *p* < 0.0001) but no effect of group, indicating there was no group differences in the percentage of AAF responding to different octaves (Supplementary Figure 1B; Supplementary Table 1). Similarly, when we compared the number of evoked spikes within each octave there was a main effect of octave (χ2 = 174, df = 4, *p* < 0.0001) but no significant effect of group (χ2 = 4.73, df = 2, *p* = 0.09), and no octave x group interaction (χ2 = 12.23, df = 8, *p* = 0.14) (Supplementary Figure 1C). Furthermore, we compared the spontaneous activity occurring in the 100 ms prior to tone onset, and observed no main effects, suggesting no group differences in spontaneous activity which could account for the observed differences in response strength (χ2 = 0.89, df = 2, *p* = 0.63). In summary, VPA-exposed rats exhibited responses to pure tones which were weaker and delayed when compared to their SAL-exposed peers. VNS-paired VPA-exposed rats exhibited a partial restoration of both response latency and response strength. These improvements were not driven by changes in the sensitivity or selectivity of AAF neurons and cannot be explained by changes in spontaneous activity or frequency representation.

#### 3.1.2 Temporal processing

**Figure 2:**
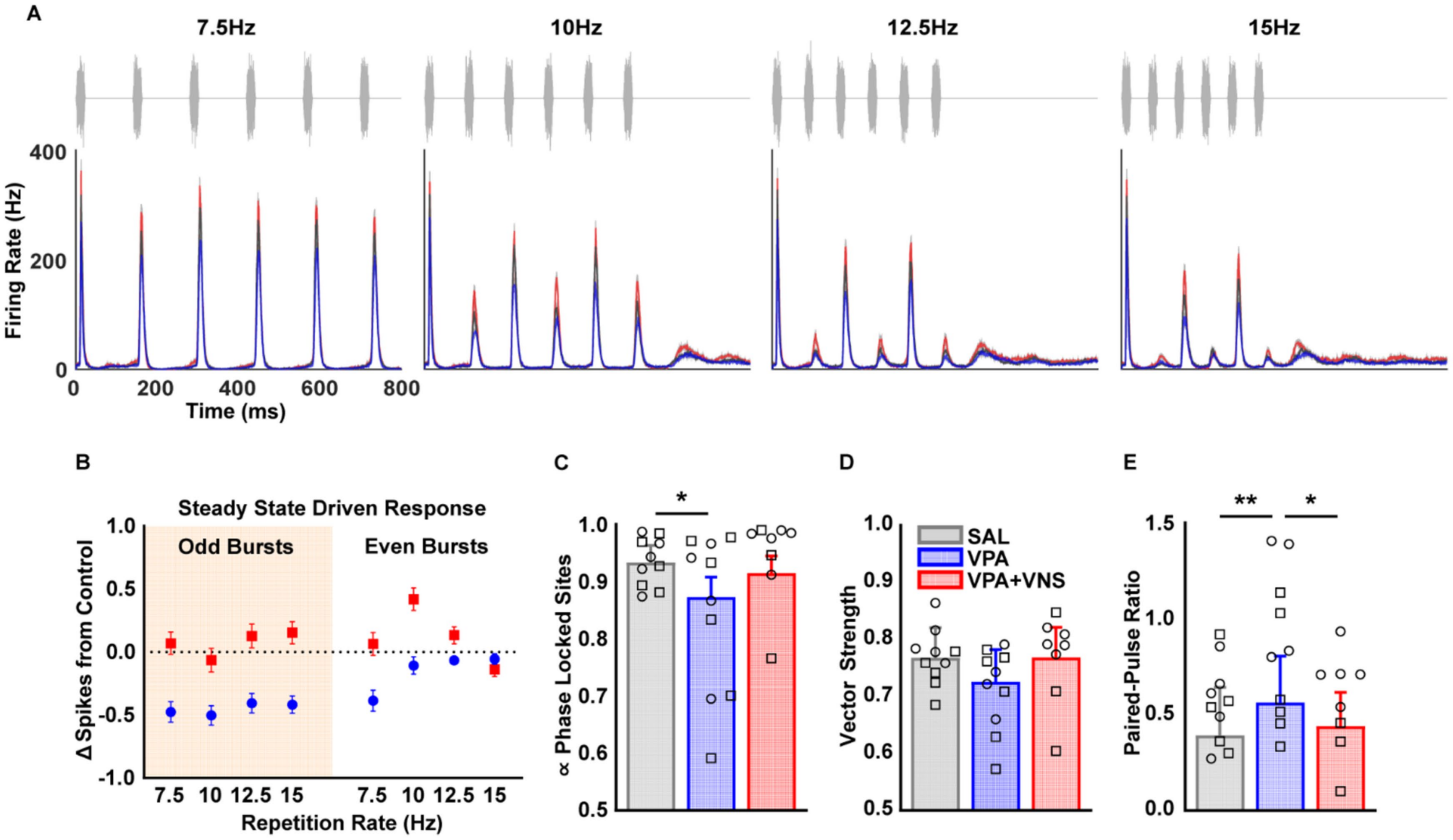
Degraded temporal processing partially restored with VNS. AAF multi-unit responses to noise burst trains with varying repetition rates (7.5–15 Hz). **(A)** Waveforms and peri-stimulus time histograms for responses to noise burst trains varying in repetition rates. **(B)** Illustration of weak evoked activity to odd bursts in the noise burst train among VPA exposed rats during steady state responses (bursts 3-6). Odd bursts (3 & 5) and even bursts (4 & 6). Data points represent mean and SEM of recording sites. **(C)** The average proportion of phase locked (Rayleigh statistic >13.8) recording sites. **(D)** The average vector strength of recording sites. **(E)** The average paired-pulse ratio (burst 2/burst 1) of recording sites. Detailed group N and information on post hoc testing is available in Table 1 and Supplementary Table 3. Bars represent mean and error bars represent standard error (SEM) of recording sites. Individual data points represent the mean across all AAF recording sites for a single animal. Shape of individual data points denote sex; squares are male and circles are female (* = *p* < 0.05, ** = *p* < 0.01).

The integration of both spectral and temporal information is necessary for complex sound processing. To assess the ability of AAF neurons to track rapid temporal changes, we recorded responses to four noise burst trains varying in repetition rate (7.5 – 15 Hz) (Figure 2A). When we compared the driven response evoked by each noise burst in each train, we observed robust effects of both group and noise burst repetition rate on driven response strength (Supplementary Figure 2; Supplementary Table 3). Post hoc comparisons revealed a compounding effect of repetition rate on firing rate, where VPA-exposed rats had significantly decreased firing to the third and fifth noise burst at the fastest repetition rate. These findings are further illustrated in Figure 2B which highlights the difference in steady state driven responses across alternating noise bursts in the train. Here VPA-exposed rats exhibit diminished steady state driven responses to odd and not even bursts in the noise train. VNS-paired VPA-exposed rats had a full restoration of this temporal processing degradation and responded significantly stronger than untreated VPA-exposed rats across all bursts in each train (Supplementary Figure 2; Supplementary Table 3). Since we observed weak driven responses among VPA-exposed rodents to alternating noise bursts in the trains, we further characterized temporal processing by assessing phase locking, vector strength, and paired-pulse ratio.

When we compared the ability of recording sites to phase lock, there was a main effect of repetition rate (χ2 = 8.147, df = 3, *p* < 0.0001), and a group x repetition rate interaction (χ2 = 12.6, df = 6, *p* = 0.04). On average across repetition rates, post hoc comparisons revealed a significant decrease in the average proportion of recording sites phase locked to noise burst trains among VPA-exposed rats compared to SAL-exposed controls. VNS-paired VPA-exposed rats exhibited a partial restoration of phase locking and were no longer significantly different from either group (Figure 2C; Table 1; Supplementary Table 3). To determine the phase synchrony of responses, we calculated the vector strength of recording sites. Here there was a main effect of repetition rate (χ2 = 950, df = 3, *p* < 0.0001), but no main effect of group (χ2 = 3.18, df = 2, *p* = 0.20, Figure 2D). Since phase synchrony and phase locking directly measure the successive probability of firing an action potential to repeating stimuli, we calculated paired-pulse ratio to determine whether the proportion of neurons firing action potentials differed between bursts in the train. There were significant main effects of group (χ2 = 8.02, df = 2, *p* = 0.01), repetition rate (χ2 = 834, df = 3, *p* < 0.0001), and group x repetition rate interaction (χ2 = 21.3, df = 6, *p* = 0.02) on paired-pulse ratio. On average across repetition rates, post hoc comparisons revealed that compared to SAL-exposed rats, VPA-exposed rats had a significantly higher probability of firing an action potential to the second burst of the noise train than the first. VNS-paired VPA-exposed rats had a full restoration of paired-pulse ratio and were no longer different from SAL controls (Figure 2E; Table 1; Supplementary Table 3). In summary, VPA-exposed rats exhibit a diminished ability to process fast repetition rate noise burst trains. This extended to a significant decrease in the proportion of phase locked neurons and a significantly higher paired-pulse ratio. These changes to temporal processing were largely restored following VNS-speech pairing. Next, we assessed whether these improvements in temporal processing led to improvements in the processing of spectrotemporally complex speech sounds.

#### 3.1.3 Response strength and latency to stop consonants

**Figure 3:**
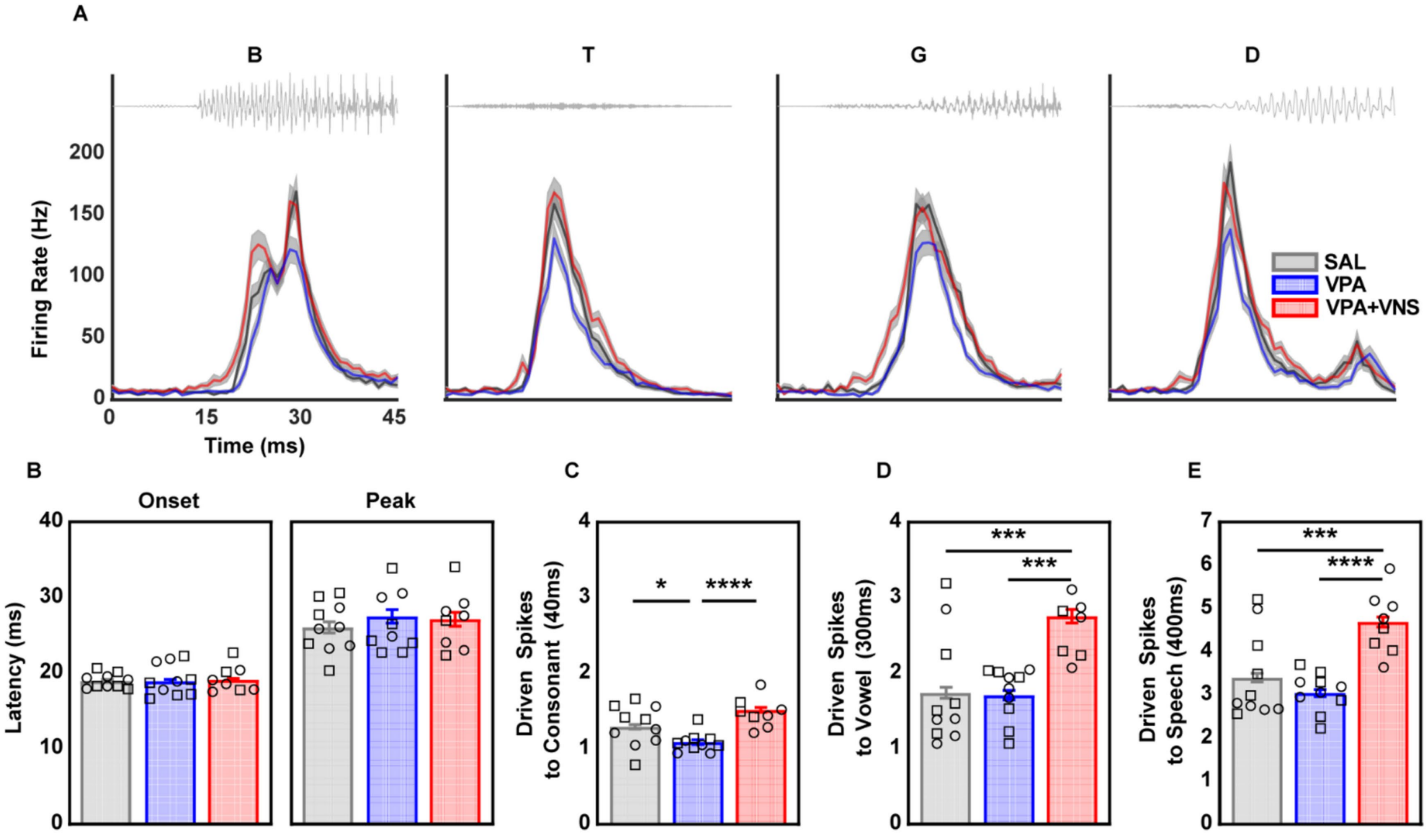
Degraded responses to stop-consonant speech sounds were fully restored with VNS. AAF multi-unit responses to stop-consonant initial speech sounds. **(A)** Waveforms and peri-stimulus time histograms for the onset of the stop consonant portion of the speech sounds. Waveforms and PSTH to entire speech sounds can be found in Supplementary Figure 3. **(B)** Onset and Peak response latency to the stop consonant portion of the speech sounds. **(C)** Driven activity during the stop consonant portion of the speech sounds. **(D)** Driven activity during the 300 ms vowel portion of speech sounds. **(E)** Driven activity during entire 400 ms speech sounds. Detailed group N and information on post hoc testing is available in Table 1. Bars represent mean and error bars represent standard error of the mean (SEM) for recording sites. Individual data points represent the mean across all sites for a single animal. Shape of individual data points denote sex; squares are male and circles are female (* = *p* < 0.05, *** = *p* < 0.001, **** = *p* < 0.0001).

Clinical literature on developmental disorders and specific language impairments often attribute speech perception difficulties to stop consonants specifically (Tallal, 2004; Hornickel et al., 2009). We grouped our speech sounds by manner of articulation and isolated our analysis to the four stop consonants presented (“b,” “t,” “g,” and “d”). We observed no main effect of group on onset (χ2 = 0.10, df = 2, *p* = 0. 49) or peak latency (χ2 = 0.96, df = 2, *p* = 0.61), suggesting no differences in response latency between groups (Figures 3A,B; Table 1). We next compared response strength driven by the onset of the consonant (1-40 ms), where there was a strong main effect of group (χ2 = 21.1, df = 2, *p* < 0.0001), and post hoc comparisons revealed significantly weaker driven activity to stop consonants among VPA-exposed rats compared to SAL controls. This deficit in response strength was completely rescued among VNS-paired VPA-exposed rats which responded significantly stronger than their untreated counterparts and were no longer different from SAL-exposed controls (Figure 3C; Table 1). Comparison of the 300 ms driven response to the vowel and the entire 400 ms stop consonant initial speech sound revealed significant main effects of group (χ2 = 19.5, df = 2, *p* < 0.0001; χ2 = 24.6, df = 2, *p* < 0.0001). Post hoc comparisons showed this was due to an increase in response strength among VNS-paired VPA-exposed rats and there was no decrease in response strength to the vowel portion of the sound for untreated VPA-exposed rats compared to SAL controls (Figures 3D,E; Table 1). These findings confirm that the rapid acoustic changes in spectral energy present in the first 40 ms of stop consonants are responsible for the degraded driven activity we observed among VPA-exposed rats. Across all speech sounds, VNS-paired VPA-exposed rats displayed a generalized increase in response strength, resulting in a full restoration of consonant processing.

#### 3.1.4 Neural discrimination of speech

**Figure 4:**
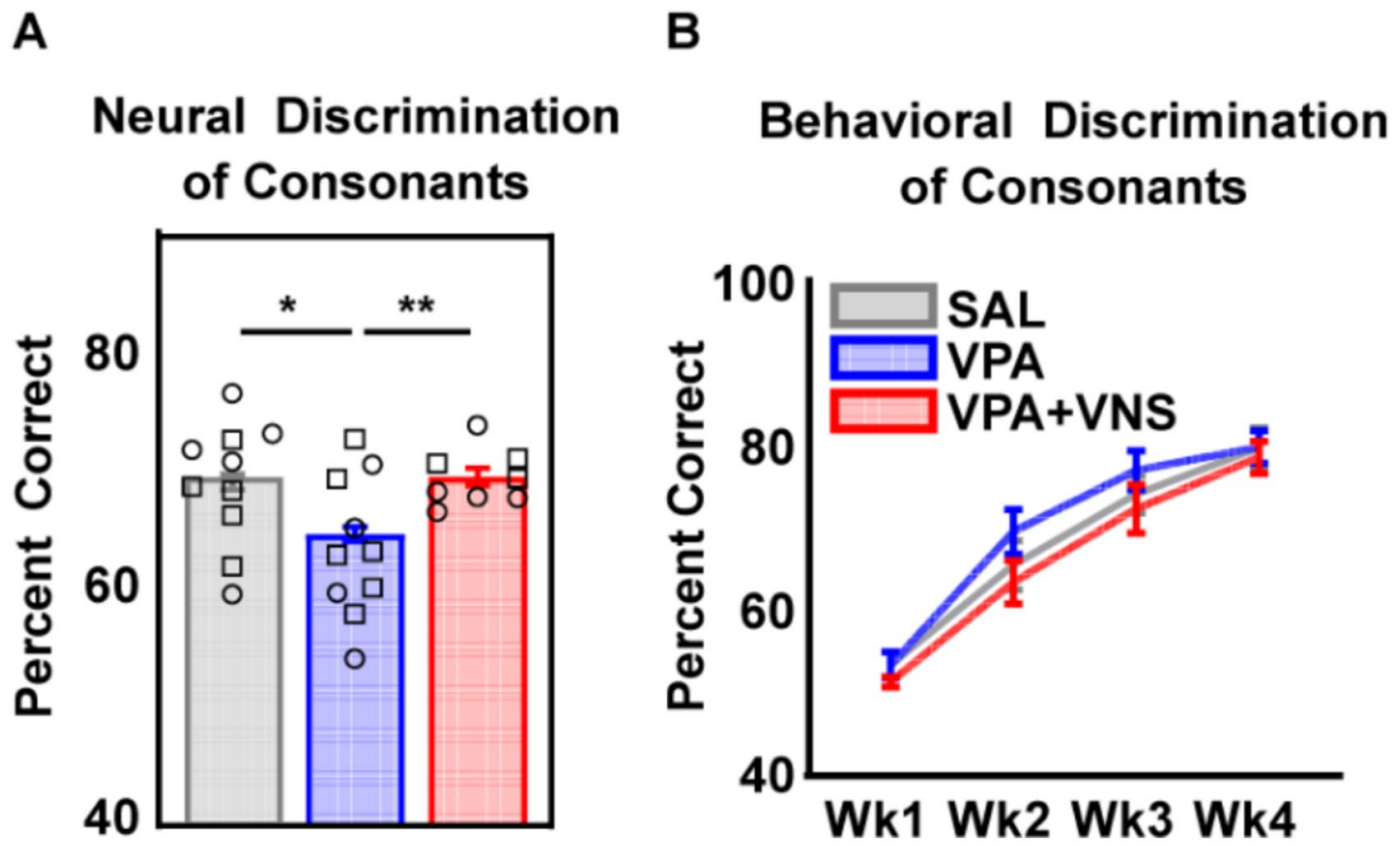
Neural discrimination of stop consonant speech sounds was fully restored with VNS. **(A)** Performance of a neural classifier trained to discriminate AAF activity patterns driven by pairs of stop consonant speech sounds. **(B)** Go/no-go behavioral discrimination of stop consonant speech sounds. Bars represent mean and error bars represent standard error of the mean (SEM) for recording sites. Individual data points represent the mean across all sites for a single animal. Shape of individual data points denote sex; squares are male and circles are female. Lines represent mean and error bars represent SEM across animals in behavior. Neural and behavioral data are from separate animals. Detailed group N and information on post hoc testing is available in Table 1 (* = *p* < 0.05, ** = *p* < 0.01).

Next, we examined neural discrimination of the same sounds, to determine whether VNS driven increases in response strength would impact neural discriminability of stop consonants. A neural classifier was trained to categorize spatiotemporal patterns of activity across 19 of 20 speech sound repeats, then asked to correctly identify the consonant presented from the final repeat of neural activity (Engineer et al., 2008). There was a significant main effect of group (χ2 = 9.19, df = 2, *p* = 0.01) on classifier performance, and post hoc comparisons revealed that recording sites from VPA-exposed rats were significantly less accurate at consonant classification compared to SAL-exposed control rats. Neural classifier discrimination was fully rescued among VNS-paired VPA-exposed rats, who were significantly different from VPA-exposed rats and no different from SAL-exposed control rats (Figure 4A; Table 1). Improved performance on the neural classifier among VNS-paired VPA-exposed rats suggests that in addition to increasing driven response strength, VNS paired with the speech sound “dad” also led to more distinct neural activity evoked by stop consonants in VPA-exposed rats.

### 3.2 Experiment 2: behavior

Behavioral discrimination of speech sounds is dependent on intact and unique neural patterns of activity (Engineer et al., 2008, 2013; Perez et al., 2013). Activity patterns can become less distinct when physiological responses to sound are weak and delayed (Engineer et al., 2014a). In a previous study, VPA-exposed rats – who also exhibit weak and delayed responses to sound – take longer to learn and perform worse than their SAL-exposed counterparts at discriminating speech sounds differing in initial consonant (Engineer et al., 2014b). Recently, success-paired VNS was shown to accelerate motor learning on a reach and grab task (Bowles et al., 2022). Here we sought to test whether success-paired VNS could accelerate learning and improve consonant discrimination for VPA-exposed rats.

#### 3.2.1 Behavioral discrimination of speech

We trained a separate set of rats to discriminate speech sounds and one group of VPA-exposed rats received success-paired VNS. Throughout the four weeks of training, we observed a significant effect of training week (ANOVA, weeks, *F* (3, 75) = 296.6, *p* < 0.0001), but no effect of treatment (ANOVA, treatment, *F* (2, 25) = 0.73, *p* = 0.49) and no week x treatment interaction (ANOVA, Weeks x Treatment, *F* (6, 75) = 1.205, *p* = 0.3133) (Figure 4B). We hypothesized that success-paired VNS may improve the generalizability of training to a variety of sound contexts, and tested the rats on additional discrimination tasks, including speech-in-noise, truncated consonants (cut to 40 ms), compressed speech, or speech sounds spoken by multiple male and female talkers. Across all tasks performance remained comparable between groups, and we failed to reveal any treatment-related differences (Supplementary Figure 4; Supplementary Table 5; additional methods in figure legend). These findings suggest that in the absence of any behavioral impairment success-paired VNS does not alter speech discrimination learning or the generalizability of training.

## 4 Discussion

For individuals with ASD, weak and delayed cortical responses to speech sounds have often been linked to receptive and expressive language impairments (Rosenhall et al., 2003; Alcántara et al., 2004; Russo et al., 2008; Otto-Meyer et al., 2018; Ramezani et al., 2019; Seif et al., 2021). Prenatal exposure to VPA causes similar language and speech processing impairments (Nadebaum et al., 2011; Christensen et al., 2013; Engineer et al., 2014a, 2014b; Anomal et al., 2015; Cheng et al., 2022; Tamaoki et al., 2024). Pairing VNS with speech sounds has been shown to drive robust changes in the auditory cortex in typically hearing rats, improving speech processing (Engineer et al., 2015; Borland et al., 2023). However, it has been unknown whether these improvements could restore the degraded cortical speech processing resulting from prenatal exposure to VPA. Our study observed degraded sound processing among VPA-exposed rats present across pure tone pips, noise bursts, and speech sounds. Across all three sound types, VNS-paired VPA-exposed rats had a partial or full restoration of sound processing. These improvements in sound processing resulted in more distinct neural activity driven by speech. However, when we tested VPA-exposed rats on their ability to behaviorally discriminate speech sounds, we failed to replicate the previously reported speech discrimination impairments, observing no behavioral differences between groups (Engineer et al., 2014b).

### 4.1 VPA auditory processing

In our study, prenatal exposure to VPA did not alter response threshold or bandwidth in AAF. This is surprising since previous research has documented significantly higher thresholds and wider bandwidths in A1 and AAF of VPA-exposed rats (Engineer et al., 2014a; Anomal et al., 2015; Cheng et al., 2022). However, recently published findings from the central nucleus of the inferior colliculus (IC) saw no change in threshold or bandwidth in VPA-exposed rats (Tamaoki et al., 2024). Similarly, previous studies have observed changes to the tonotopic organization of A1 and frequency dependent changes in firing rate (Anomal et al., 2015; Cheng et al., 2022). In our study we saw no differences in tonotopic organization or tone evoked responses across frequency. Previously it was reported that in A1, tone evoked responses are significantly faster among VPA-exposed rats (Anomal et al., 2015). In IC it was reported that there were no changes to tone evoked response latency (Tamaoki et al., 2024). However, in our study VPA-exposed rats exhibited a significant delay in the onset and peak of tone evoked responses. Interestingly, clinical literature on pure tone processing in ASD appears to mirror these highly variable findings, with some studies attributing enhanced pitch perception among children with ASD to shorter response latencies in a mismatch negativity paradigm (Gomot et al., 2002). Utilizing similar techniques, others report individuals with ASD as having no change in response latency (Ceponiene et al., 2003) or an increase in response latency (Jansson-Verkasalo et al., 2003).

Another clinical parallel is in intensity coding, where individuals with ASD appear to have weaker amplitude responses to pure tones across sound intensity (Bruneau et al., 1999, 2003). The same has been reported in AAF but not IC of VPA-exposed rats who responded significantly weaker across all sound intensities (Engineer et al., 2014a; Tamaoki et al., 2024). Our study replicated these findings, observing weaker tone evoked responses across sound intensities among VPA-exposed rats.

Temporal processing has long been used to gauge the function of the auditory network (Ribaupierre et al., 1972; Merzenich et al., 1992; Eggermont and Smith, 1995; Kilgard and Merzenich, 1998). Integration of rapid auditory stimuli appear to predict many components of language for individuals with ASD (Demopoulos et al., 2023). Previous studies characterizing temporal processing among individuals with ASD have observed a diminished ability to integrate rapidly presented stimuli and an increased interstimulus interval necessary to distinguish temporal order (Kwakye et al., 2011) or detect stimulus gaps (Foss-Feig et al., 2017). These impairments in detecting minute temporal shifts may contribute to the degraded speech processing among individuals with ASD. Studies on temporal processing in rodents typically rely on neural and not behavioral responses to characterize function. Recording responses to a 10 Hz noise burst train or tone trains at various presentation rates (10-20 pips per second), researchers have repeatedly found lower vector strength among VPA-exposed rodents, suggesting poorly synchronized neural responses (Engineer et al., 2014a; Cheng et al., 2022). Our study observed VPA-exposed rats to exhibit lower, but not significantly different vector strength when compared to their SAL-exposed peers. They did, however, exhibit other measures of impaired temporal processing including a lower proportion of phase locked recording sites and an increased paired-pulse ratio. These findings also suggest poor neural synchrony and some abnormal synaptic function consistent with existing research (Anomal et al., 2015; Cheng et al., 2022).

Contrary to our hypothesis and previously published research, we observed no group differences in onset or peak response latency to speech. This was surprising since responses to tones were delayed, and delayed responses to speech are commonly reported among VPA-exposed rats (Engineer et al., 2014a, 2014b; Tamaoki et al., 2024), among children prenatally exposed to VPA (Loucas et al., 2008; Nadebaum et al., 2011), and those diagnosed with ASD (Russo et al., 2005, 2009). Although delayed responses to speech among children with ASD are common, there appears to be some circumstances where children with ASD exhibit no delay in responses (Whitehouse and Bishop, 2008), or they respond faster than their typically developing counterparts (Yoshimura et al., 2016). In our study, VPA-exposed rats responded significantly weaker to the onset of stop consonants. Diminished response strength to the onset of consonants and stop consonants specifically has been previously reported (Engineer et al., 2014a, 2014b; Tamaoki et al., 2024). This is consistent with clinical literature on developmental language disorders and ASD where the processing of stop consonants is particularly difficult because of their rapid spectrotemporal shifts (Tallal, 2004; Hornickel et al., 2009). Since we observed degraded temporal processing in response to noise burst trains, it is not surprising that responses would also be diminished to stop consonants which rely on precisely timed responses for neural coding (Engineer et al., 2008; Perez et al., 2013). In fact, when we trained a neural classifier to distinguish between pairs of stop consonants, VPA-exposed rodents performed significantly worse, suggesting that the patterned activity evoked by stop consonant speech sounds is less distinct. This is consistent with previous research on VPA-exposed rats in both the IC and AAF (Engineer et al., 2014a, 2014b; Tamaoki et al., 2024).

It has been established that similar patterns of activity are more difficult to discriminate behaviorally (Engineer et al., 2008), and when tested, VPA-exposed rats previously exhibited deficits in the behavioral discrimination of speech sounds (Engineer et al., 2014b). However, in our study there was no difference in discrimination behavior of VPA-exposed rats and their SAL-exposed counterparts. One possible explanation for the failure to replicate is that changing the task kinematics from lever press to nose poke may have masked a more complex impairment in sensorimotor integration. Other potential explanations include differences in the training timeline between studies, and potential inadvertent differences in the timing of the initial prenatal VPA injection (12.5 days post conception). We had hypothesized that changing the spectral components of the sound by nesting them in background noise or compressing the sound to increase the temporal processing demand should make them more difficult to discriminate (Engineer et al., 2013), but additional tasks with more spectrotemporally complex sounds failed to reveal any group differences. It is possible that any sound processing impairments initially present in VPA-exposed rats were ameliorated with training (Engineer et al., 2014b), resulting in no observable impairments in subsequent tasks with more complex sounds. Interestingly, there appears to be some context or content specific situations in which individuals with ASD perform auditory feature discrimination at the same level or better than their typically developing peers (Gomot et al., 2002; Alcántara et al., 2004; Groen et al., 2009; Jones et al., 2009). Although we failed to replicate a previously reported deficit in speech sound discrimination among VPA-exposed rats, there may be tasks which VPA-exposed rats are reliably impaired at. It is also possible that with a larger sample size perturbations to typical behavior may appear, like the subtle changes in attention that some have reported (Chomiak et al., 2014).

### 4.2 VNS auditory processing

Studies assessing speech-paired VNS in typically hearing rodents report improvements in response latency, increases in response strength to tones and speech, and more unique patterns of activity driven by speech (Engineer et al., 2015; Borland et al., 2023). In our study we observed that this remained true even when sound processing was degraded through prenatal exposure to VPA. Across sound types, sound-paired VNS led to a partial or complete restoration of processing for VPA-exposed rats. VNS-paired VPA-exposed rats exhibited a partial restoration of response latency, no longer exhibiting delayed onset or peak activity. VNS-paired VPA-exposed rats had a partial restoration of intensity coding, responding significantly stronger than untreated VPA-exposed rats, but in most cases still significantly lower than SAL-exposed rats. Furthermore, responses to tones were improved without altering neuron tuning or decreasing threshold. This suggests that sound-paired VNS can be used to improve receptive sound processing without having off-target effects on neuron sensitivity or selectivity.

Our study expands on previous literature by assessing temporal processing after speech-paired VNS. We saw that speech-paired VNS led to a complete restoration of temporal processing. VNS-paired VPA-exposed rats had significantly stronger responses across all bursts in the train, an increased number of phase locked recording sites, and a complete restoration of paired-pulse ratio.

Our study continues to expand on previously published literature by showing that speech-paired VNS led to a complete restoration of speech processing among rodents prenatally exposed to VPA. VNS-paired VPA-exposed rats exhibited a complete restoration of response strength to the onset of stop consonant speech sounds and had a general increase in response strength to the 300 ms vowel and the entire 400 ms speech sound. Furthermore, these increases in response strength led to more unique patterns of activity which were significantly more distinct than response patterns of untreated VPA-exposed rats.

Improvements in neural discrimination of speech should lead to behavioral improvements in the discrimination of speech (Engineer et al., 2008). However, we observed no effects of success-paired VNS on speech discrimination behavior. Recent studies suggest that a behavioral deficit is necessary to observe any VNS driven improvements (Carroll et al., 2024). The slight change in timing between sound-paired and success-paired VNS may also influence the efficacy of paired VNS. It has been shown in the motor and visual cortex that slight changes in the timing of neuromodulator release relative to synaptic activity can determine the effect it has on learning and cortical plasticity (He et al., 2015; Bowles et al., 2022). While this remains unknown for the auditory cortex, previous studies have observed transient effects of paired neuromodulator release on neuron tuning (Froemke et al., 2007; Martins and Froemke, 2015). In a study that investigated pairing locus coeruleus stimulation with tones, it was shown that delivering stimulation during behavior impaired discrimination, but decoupling pairings from behavior by hours accelerated perceptual learning (Martins and Froemke, 2015; Martin et al., 2024). In our study we observed no deleterious effects of success-paired VNS on speech discrimination behavior. It remains unknown whether success-paired VNS leads to changes in the cortical representation of speech or alters receptive field properties.

### 4.3 Limitations

Although this study included animals of both sexes, the groups were not powered to detect sex differences. Therefore, we cannot rule out sex as a contributing factor to our results, despite observing no within group sex differences.

### 4.4 Clinical implications

In our study, speech-paired VNS drove widespread improvements in sound processing, partially or fully restoring the processing of tones, noise burst trains, and speech. These physiological changes improved the neural encoding of sound, and as a correlate, our study observed more unique patterns of activity driven by speech after VNS pairing. Despite the improvements in neural sound processing, our study did not see VNS driven improvements in speech sound discrimination. Although this is likely due to the lack of behavioral impairment among VPA-exposed rats, documenting a clear behavioral correlate of the improvements to neural sound processing is an important step in the clinical translatability of sound-paired VNS. Fortunately, VNS is already FDA approved for the treatment of drug-resistant epilepsy, and some children with ASD are already implanted with pulse generators and regularly receive VNS. In a recent observational study of ten children with ASD who received VNS for the treatment of their epileptic seizures, researchers reported a significant improvement in language as measured by the Autism Behavior Checklist (Fumagalli Marteleto and Marcondes Pedromonico, 2005; Wang et al., 2022). All ten children had improvements in language and two had complete remission of language related ASD symptomatology with less than one year of treatment (Wang et al., 2022). Although this study has a small sample size and does not use a standardized assessment of language (like the Clinical Evaluation of Language Fundamentals) (Paslawski, 2005), these findings offer hope for the implementation of VNS as an adjunct to traditional ASD interventions. Supplementing therapy by pairing VNS with important speech sounds could improve the efficacy of treatment and increase the number of people who make meaningful improvements from therapy.

Timing of intervention appears to influence outcomes for children with ASD, with those starting earlier making more meaningful improvements (Anderson et al., 2014; Towle et al., 2020). Although the median age for childhood diagnosis and treatment of ASD is between 4-5 years old, outcomes are still better for those diagnosed and treated earlier (Anderson et al., 2014; van’t Hof et al., 2021). Since our study only assesses intervention in adulthood, additional studies considering the clinical implementation of VNS will need to determine how early intervention influences VNS efficacy.

## Data Availability

The raw data supporting the conclusions of this article will be made available by the authors, without undue reservation.
