# Supplementary material for: Speech paired vagus nerve stimulation restores neural sound processing in a rat model of autism
Source: Front Neurosci. 2025 Jun 13;19:1600024. doi: 10.3389/fnins.2025.1600024 (PMC12202670; doi:10.3389/fnins.2025.1600024)
Supplement: Supplementary file 1 [file Supplementary_file_1.docx]

Supplementary Material


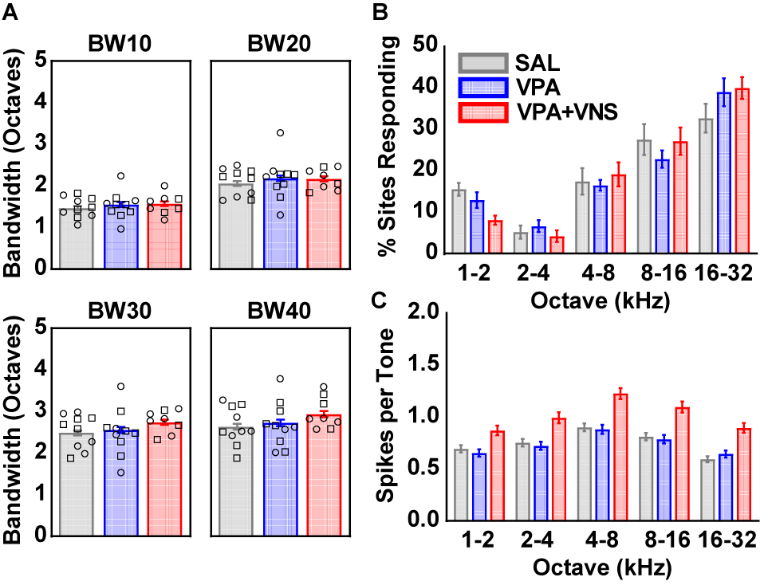


**SFigure 1: Additional receptive field properties.** Anterior Auditory Field multi-unit responses to pure tone pips. Bars represent mean and error bars represent standard error (SEM) of the mean for recording sites. Individual data points represent mean across all sites for a single animal. Shape of individual data points denote sex; squares are male and circles are female. (A) Bandwidth (in octaves) of neural tuning 10, 20, 30, & 40 dB above threshold. (B) The percentage of recording sites responding in octave bins. (C) The average number of spikes evoked per tone, in five 1-octave tone frequency bins. Detailed group N and information on post hoc testing available in STable 1.


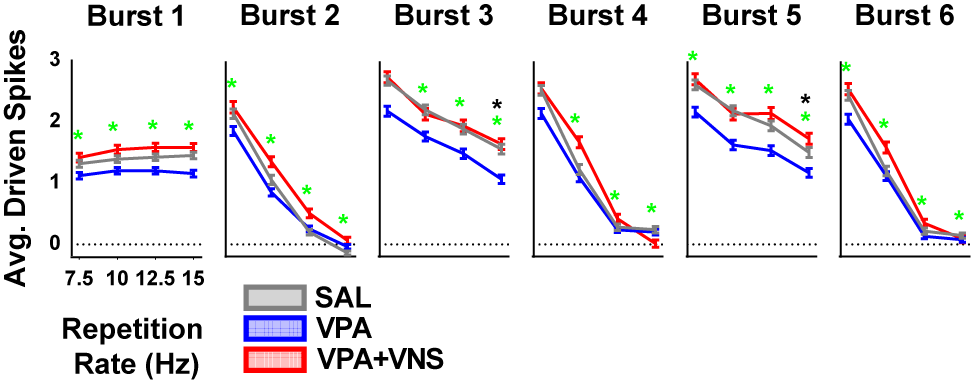


**SFigure 2: Average Driven Firing Rate for Each Burst in Each Noise Burst Train**. Lines represent mean and error bars represent standard error of the mean (SEM) for recording sites. There were significant main effects of group (χ2= 8.70, df=2, p=0.01), repetition rate (χ2= 4420.74, df=3, p<0.0001), and burst (χ2= 2787.45, df=5, p<0.0001). Post hoc comparisons shown in STable 3 highlight the between group differences across all bursts in the noise burst train. Plots are the average driven response to each noise burst in the train by repetition rate. Green asterisks are a significant difference between VPA and VNS treated VPA exposed rats, black asterisks are significant differences between SAL exposed controls and VPA exposed rats.


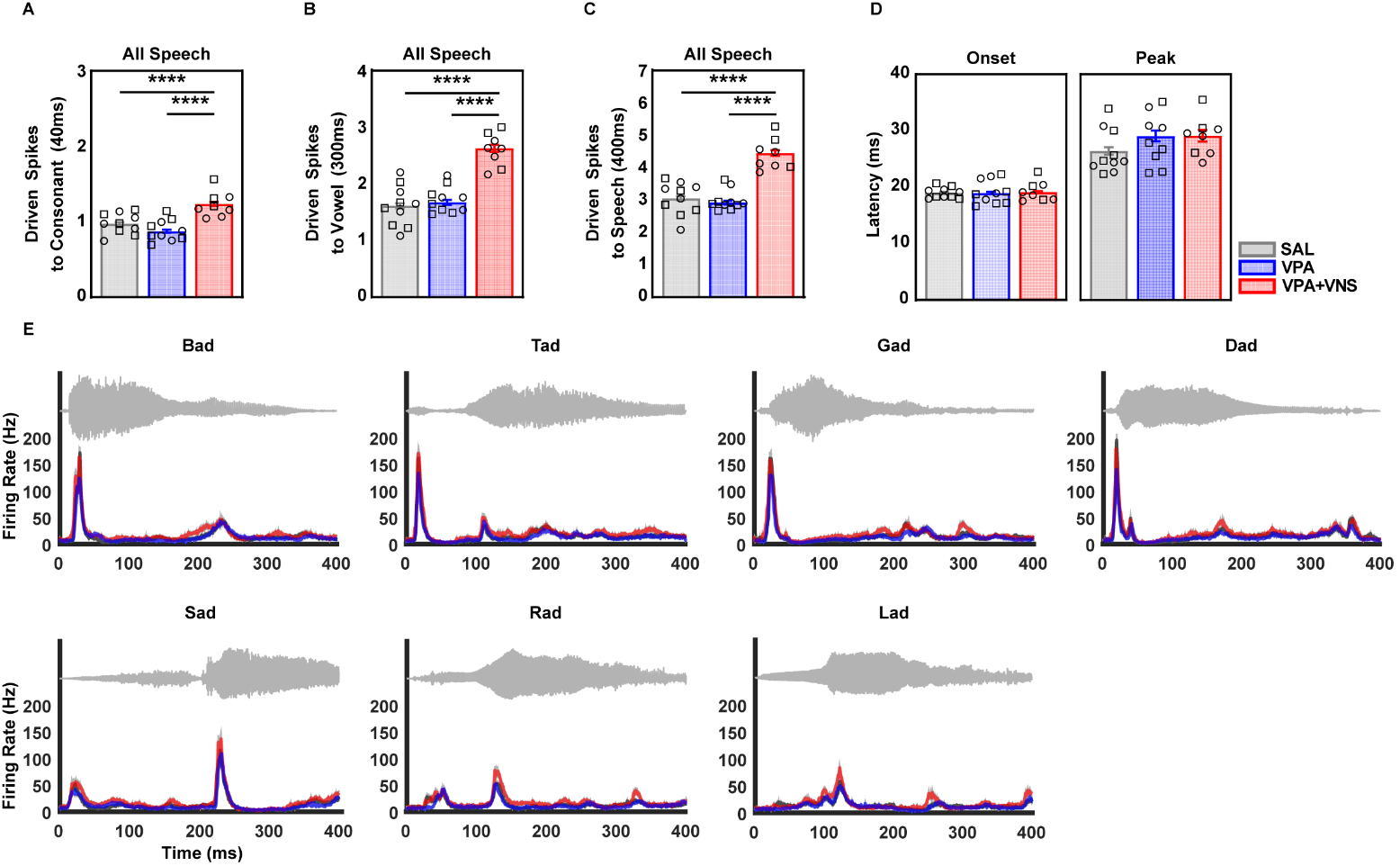


**SFigure 3: Additional response data for all speech sounds played.** Anterior Auditory Field multi-unit responses to all speech sounds played during recordings (“B”, “G”, “T”, “D”, “S”, “R”, & “L”). Bars represent mean and error bars represent standard error (SEM) of the mean for recording sites. Individual data points represent mean across all sites for a single animal. Shape of individual data points denote sex; squares are male and circles are female. (A-C) Driven responses to the consonant onset (40ms), vowel (300ms), and entire (400ms) speech sounds. (D) Onset and Peak response latency averaged across the seven speech sounds. (E) Waveforms and peri-stimulus time histograms for all speech sounds played during recordings. Detailed group N and information on post hoc testing available in STable 4. ** = p<0.01, **** = p<0.0001.


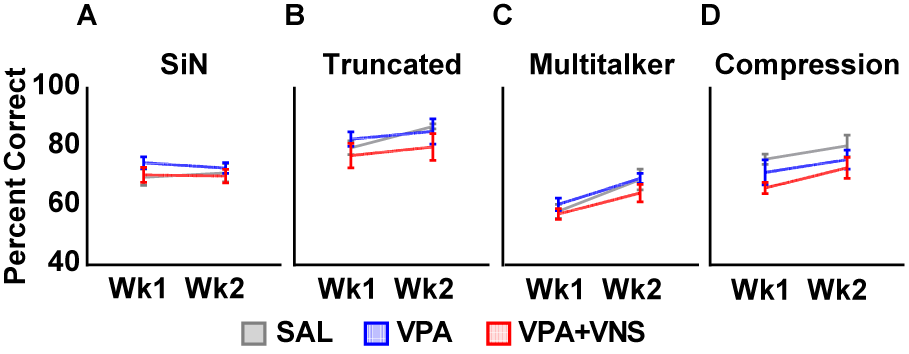


**SFigure 4: Additional go/no-go sound discrimination tasks.** All four panels represent performance across additional go/no-go speech sound discrimination tasks that animals were tested on after the consonant discrimination task reported in Figure 4. Lines denote mean and standard error of the mean (SEM) during behavior binned by week. Two-way repeated measures ANOVA results for each figure are available in STable 5. No significant treatment x week interactions were observed so posthoc testing was not conducted. (A) Percent correct for a stop consonant discrimination task where speech sounds (60dB) are masked by varying levels of background noise (0, 48, 54, 72db). There were no differences in group performance across any noise level, so results shown here are averaged across noise levels for simplicity. (B) Percent correct for a stop consonant discrimination task where speech sounds are cut to only the onset of the consonant (1-40ms). (C) Percent correct on a “dad” vs “tad” discrimination task where each sound is voiced by three different male and female speakers. No group differences were observed across speakers, so results shown here are averaged across speakers. (D) Percent correct on a “dad” vs “tad” discrimination task where each speech sound is compressed in ten 10% steps so that a 50% compression is ½ the original sound duration. No group differences were observed across any compression step, so the results shown here are averaged across compression step for simplicity. Groups are n=3-5 for all tasks.


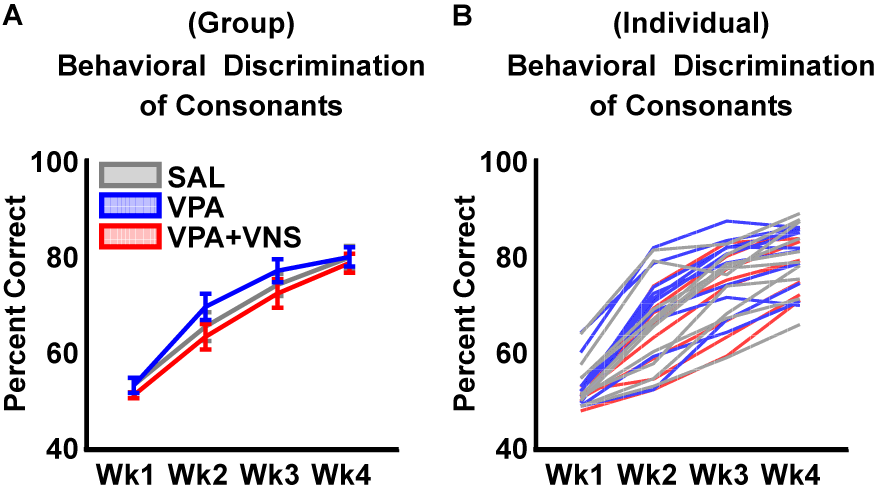


**SFigure 5: Comparison of group and individual performance on consonant discrimination.** Go/no-go behavioral discrimination of stop consonant speech sounds. (A) Mean group performance with SEM across animals. (B) Individual animal performance. Detailed group N and information on post hoc testing is available in Table 1.

**SUPPLEMENTAL TABLES**

Table 1, STable 1, STable 2, & STable 3 are longer than a page and attached as separate excel sheets.

**Supplemental Table 4: Model and Estimated Marginal Means for Supplemental Figure 3.** Bolded pvalues are significant.

| **Figure** | **Model** | **Group**  **(n sites / n animal)** | **Back transformed estimated marginal mean** | **Lower CI** | **Upper CI** | **SE** | **Contrast** | **Pvalue** |
| --- | --- | --- | --- | --- | --- | --- | --- | --- |
| SFigure3a | GLMM: Spikes ~ Group + Sex + Group * Sex + (1 \| Animal/Channel), family = gaussian(link="log") | Saline (n=357/10) | 0.94 | 0.87 | 1.02 | 0.03 | SAL / VPA | 0.14 |
|  |  | VPA (n=330/10) | 0.85 | 0.78 | 0.92 | 0.03 | SAL / VPA+VNS | **<0.0001** |
|  |  | VNS (n=287/8) | 1.21 | 1.12 | 1.32 | 0.05 | VPA / VPA+VNS | **<0.0001** |
| SFigure3b | GLMM: Spikes ~ Group + Sex + Group * Sex + (1 \| Animal/Channel), family = tweedie(link="log") | Saline (n=357/10) | 1.59 | 1.45 | 1.75 | 0.07 | SAL / VPA | 0.52 |
|  |  | VPA (n=330/10) | 1.71 | 1.55 | 1.89 | 0.08 | SAL / VPA+VNS | **<0.0001** |
|  |  | VNS (n=287/8) | 2.62 | 2.36 | 2.9 | 0.13 | VPA / VPA+VNS | **<0.0001** |
| SFigure3c | GLMM: Spikes ~ Group + Sex + Group * Sex + (1 \| Animal/Channel), family = tweedie(link="log") | Saline (n=357/10) | 2.99 | 2.78 | 3.21 | 0.11 | SAL / VPA | 0.93 |
|  |  | VPA (n=330/10) | 2.93 | 2.71 | 3.16 | 0.11 | SAL / VPA+VNS | **<0.0001** |
|  |  | VNS (n=287/8) | 4.4 | 4.06 | 4.77 | 0.18 | VPA / VPA+VNS | **<0.0001** |
| SFigure3d(a) | GLMM: Onset ~ Group + Sex + Group * Sex + (1 \| Animal/Channel), family= gaussian (link="log") | Saline (n=357/10) | 18.8 | 18 | 19.7 | 0.44 | SAL / VPA | 0.91 |
|  |  | VPA (n=330/10) | 19.1 | 18.2 | 20 | 0.46 | SAL / VPA+VNS | 0.82 |
|  |  | VNS (n=287/8) | 19.2 | 18.2 | 20.3 | 0.52 | VPA / VPA+VNS | 0.97 |
| SFigure3d(b) | GLMM: Peak ~ Group + Sex + Group * Sex + (1 \| Animal/Channel), family= gaussian (link="log") | Saline (n=357/10) | 24.6 | 23.1 | 26.3 | 0.81 | SAL / VPA | 0.17 |
|  |  | VPA (n=330/10) | 26.9 | 25.1 | 28.8 | 0.94 | SAL / VPA+VNS | 0.42 |
|  |  | VNS (n=287/8) | 26.2 | 24.3 | 28.3 | 1.00 | VPA / VPA+VNS | 0.89 |

**Supplemental Table 5: ANOVA Table for Supplemental Figure 4.** Bolded pvalues are significant

| **Figure** | **Model** | **Anova Table** | **Pvalue** |
| --- | --- | --- | --- |
| SFigure4a | Two-way RM ANOVA: Speech in Noise | Weeks x Treatment | 0.0623 |
|  |  | Weeks | 0.5904 |
|  |  | Treatment | 0.601 |
|  |  | Animal | **<0.0001** |
| SFigure4b | Two-way RM ANOVA: Truncated Consonants | Weeks x Treatment | 0.2804 |
|  |  | Weeks | **0.007** |
|  |  | Treatment | 0.5457 |
|  |  | Animal | **0.0004** |
| SFigure4c | Two-way RM ANOVA: Multiple Voicers | Weeks x Treatment | 0.5444 |
|  |  | Weeks | **<0.0001** |
|  |  | Treatment | 0.4512 |
|  |  | Animal | **0.0116** |
| SFigure4d | Two-way RM ANOVA: Compressed Consonants | Weeks x Treatment | 0.7886 |
|  |  | Weeks | **0.0128** |
|  |  | Treatment | 0.1625 |
|  |  | Animal | **0.0332** |

**Supplemental Table 6: *X*^2^ Chi Square test results for Figures 1-4.**

| **Figure** | **Main Effect** | ***X*^2^ (Chi Square)** |
| --- | --- | --- |
| Figure 1a(a) | Group | χ2= 6.41 |
| Figure 1a(b) | Group | χ2= 6.79 |
| Figure 1b | Group | χ2= 105 |
| Figure 1b | Intensity | χ2= 6017 |
| Figure 1b | Group x Intensity | χ2= 194 |
| Figure 1c | Group | χ2= 6.48 |
| Figure 2c | Repetition Rate | χ2= 8.147 |
| Figure 2d | Group x Repetition Rate | χ2= 12.6 |
| Figure 2e | Group | χ2= 8.02 |
| Figure 2e | Repetition Rate | χ2= 834 |
| Figure 2e | Group x Repetition Rate | χ2= 21.3 |
| Figure 3b(a) | None | χ2= 0.10 |
| Figure 3b(b) | None | χ2= 0.96 |
| Figure 3c | Group | χ2= 21.1 |
| Figure 3d | Group | χ2= 19.5 |
| Figure 3e | Group | χ2= 24.6 |
| Figure 4a | Group | χ2= 9.19 |

**
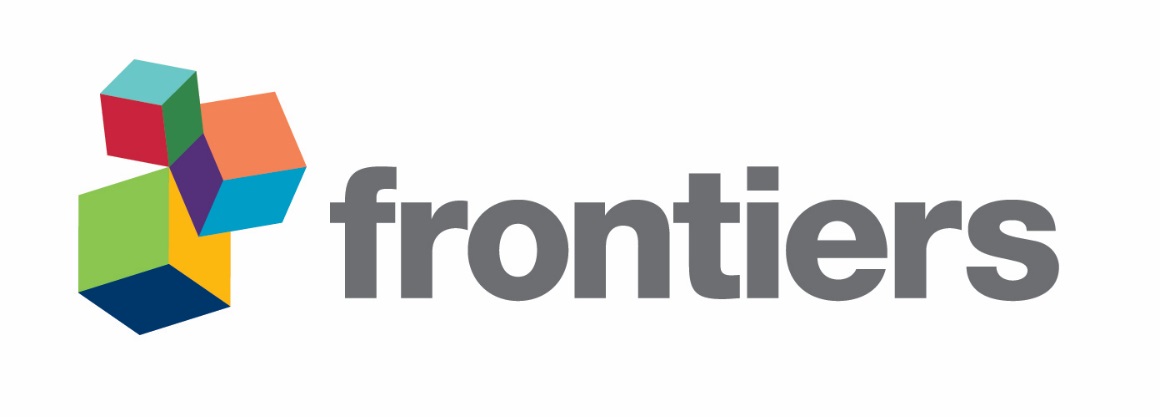
**
